# Structure of the Peptidoglycan Synthase Activator LpoP in *Pseudomonas aeruginosa*

**DOI:** 10.1016/j.str.2020.03.012

**Published:** 2020-06-02

**Authors:** Nathanael A. Caveney, Alexander J.F. Egan, Isabel Ayala, Cédric Laguri, Craig S. Robb, Eefjan Breukink, Waldemar Vollmer, Natalie C.J. Strynadka, Jean-Pierre Simorre

**Affiliations:** 1 Department of Biochemistry and Molecular Biology and Centre for Blood Research, The University of British Columbia, Vancouver V6T 1Z3 BC, Canada; 2 Centre for Bacterial Cell Biology, Biosciences Institute, Newcastle University, Richardson Road, Newcastle upon Tyne, NE2 4AX, UK; 3 University of Grenoble Alpes, CNRS, CEA, IBS, 38000 Grenoble, France; 4 Department of Membrane Biochemistry and Biophysics, Utrecht University, Utrecht 3584 CH, The Netherlands

**Keywords:** structure, bacterial cell wall, *Pseudomonas*, enzyme, catalysis, PBP activator, NMR, crystallography, lipoprotein

## Abstract

Peptidoglycan (PG) is an essential component of the bacterial cell wall and is assembled from a lipid II precursor by glycosyltransferase and transpeptidase reactions catalyzed in particular by bifunctional class A penicillin-binding proteins (aPBPs). In the major clinical pathogen *Pseudomonas aeruginosa*, PBP1B is anchored within the cytoplasmic membrane but regulated by a bespoke outer membrane-localized lipoprotein known as LpoP. Here, we report the structure of LpoP, showing an extended N-terminal, flexible tether followed by a well-ordered C-terminal tandem-tetratricopeptide repeat domain. We show that LpoP stimulates both PBP1B transpeptidase and glycosyltransferase activities *in vitro* and interacts directly via its C terminus globular domain with the central UB2H domain of PBP1B. Contrary to the situation in *E. coli*, *P. aeruginosa* CpoB does not regulate PBP1B/LpoP *in vitro*. We propose a mechanism that helps to underscore similarities and differences in class A PBP activation across Gram-negative bacteria.

## Highlights

• Structural organization of LpoP has been resolved
• ^*Pa*^PBP1B is directly stimulated by LpoP but not by ^*Pa*^CpoB *in vitro*
• LpoP likely interacts between the GTase and UB2H domains of ^*Pa*^PBP1B

## Graphical Abstract

**Figure:**
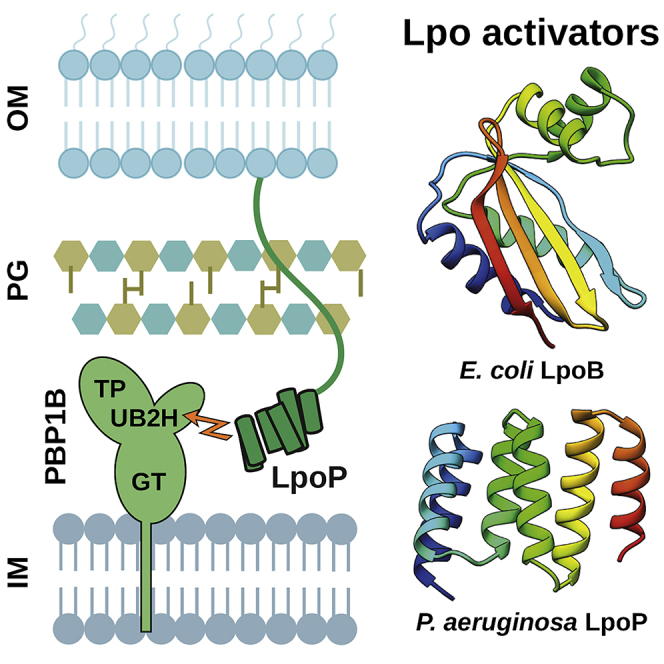

## Teaser

Caveney et al. report the structure and domain organization of LpoP and its interaction site with PBP1B from *P. aeruginosa*. They show that LpoP has an intrinsically disordered N-terminal extension, followed by a globular domain, which is different from the structure of the globular domain of *E. coli* LpoB. They show that this domain specifically interacts with the UB2H domain of ^*Pa*^PBP1B.

## Introduction

The biosynthesis of the bacterial cell wall is an excellent target for antibacterial therapy, as exemplified by the clinical action of β-lactam antibiotics, which inhibit one of the final steps in the pathway of peptidoglycan (PG) biosynthesis. PG is comprised of polymerized glycan strands of alternating, β-1,4-linked N-acetylglucosamine and N-acetylmuramic acid. Adjacent strands are covalently linked by short peptides protruding from the N-acetylmuramic acid moieties to form an extended mesh-like macromolecule called the sacculus, which surrounds the cytoplasmic membrane (Caveney et al., 2018, Typas et al., 2012). PG plays a crucial structural role in the bacterial cell envelope, and defects in PG typically result in cell lysis within the natural environs of the bacteria (Caveney et al., 2018, Egan et al., 2017).

PG biosynthesis initiates in the cytoplasm and leads to production of the membrane-localized glycopeptide precursor, lipid II, which is flipped to the external leaflet of the cytoplasmic membrane for the polymerization of glycan strands and crosslinking of peptides by PG synthases. Class A penicillin-binding proteins (aPBPs) are the critical and principal PG synthase for proper cell wall assembly and cell integrity in most bacteria where they have been investigated (Cho et al., 2016). These bifunctional enzymes perform, in distinct active sites, both the glycosyltransferase (GTase) activity, which polymerizes glycan strands from lipid II, and a DD-transpeptidase (TPase) activity, which cleaves a D-Ala^4^-D-Ala^5^ peptide bond of the acyl donor and transfers the D-Ala^4^ carbonyl to the primary amine of a diaminopimelic acid residue on the acceptor peptide. The DD-TPase activity is blocked by β-lactam antibiotics, which irreversibly acylate the conserved catalytic serine nucleophile (Caveney et al., 2018).

Over the last years, it became evident that the dual activities of some class A PBPs are differentially regulated by cognate outer membrane-anchored lipoprotein activators, LpoA (mainly TPase) and LpoB (GTase and TPase) (Typas et al., 2010, Lupoli et al., 2014, Egan et al., 2018). Moreover, *Escherichia coli* subjects the LpoB-mediated stimulation of PBP1B to regulation by CpoB, which functions together with members of the Tol system to coordinate outer membrane constriction with septal PG synthesis (Gray et al., 2015). These regulators have all been identified and characterized, at least in part, in *E. coli* with recent work providing insights into their structures, interface with the PBP, and mechanisms of PBP activation (Egan et al., 2014, Egan et al., 2018, Jean et al., 2014, Kelley et al., 2019, King et al., 2014, Markovski et al., 2016). These studies suggest that activators bind to a non-catalytic docking domain (termed the UB2H domain in PBP1B) causing conformational changes that ultimately affect active site residues and presumably consequent catalytic turn over (Markovski et al., 2016, Egan et al., 2018). The significant nosocomial pathogen *Pseudomonas aeruginosa* also has a suggested lipoprotein activator of its cognate PBP1B, termed LpoP, which is distinct in primary sequence to the *E. coli* LpoB (Greene et al., 2018). Importantly, LpoP was shown to be essential for *Pseudomonas* PBP1B function in the cell, and additional preliminary investigation to unravel the mechanism of activation was performed (Greene et al., 2018), However, the structure of LpoP remained unknown as well as experimental demonstration of the direct activation of PBP1B by LpoP (Greene et al., 2018).

Here, we show that LpoP shares general domain organization with LpoB, with both having a long, intrinsically disordered N-terminal region and a structured C-terminal region. Our crystal structure of the ordered C-terminal region revealed a tandem-tetratricopeptide repeat (tandem-TPR) structure. We probed the role of LpoP in activating *P. aeruginosa* PBP1B (^*Pa*^PBP1B) and show that LpoP interacts with ^*Pa*^PBP1B and stimulates its GTase and TPase activities *in vitro*. In addition, we show that *P. aeruginosa* CpoB (^*Pa*^CpoB) does not regulate ^*Pa*^PBP1B/LpoP *in vitro*, contrary to its role in *E. coli*. To further dissect the ^*Pa*^PBP1B-LpoP interaction, we show that the UB2H domain of ^*Pa*^PBP1B interacts with the C terminus of LpoP and use NMR to probe this interface. We propose a mechanism for the activation of PBP1B in *P. aeruginosa* that helps to understand similarities and differences in class A PBP activation across Gram-negative bacteria.

## Results

### Structural Organization of Full-Length LpoP

**Figure 1:**
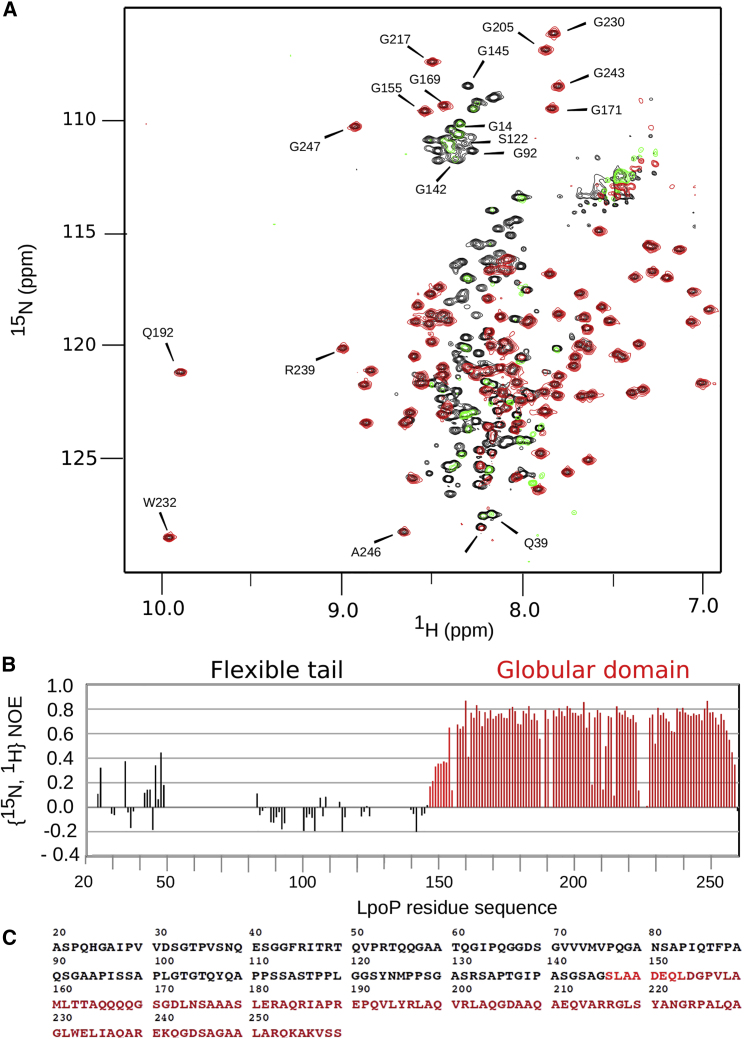
LpoP Has an Intrinsically Disordered N-Terminal Domain and a C-Terminal Globular Domain (A) Overlay of the ^1^H-^15^N heteronuclear NOE recorded with and without initial proton saturation. Saturated experiment is plotted in red and green for positive and negative contour, respectively, whereas the reference experiment recorded without ^1^H saturation is plotted in black. Both 2D ^1^H-^15^N experiments were collected on a 16.4-T NMR spectrometer at 25°C. Protein samples were prepared at 0.2 mM in 30 mM HEPES, 200 mM KCl buffer (pH 7.5). (B) Heteronuclear ^1^H-^15^N-NOE values calculated from the intensity ratio between the saturated and reference experiments. Regions with sequential positive ^1^H-^15^N-NOE values are colored in red and correspond to a structured domain. Errors in the intensity ratio were calculated from the signal-to-noise ratio of the NMR signal in each spectrum. The numbering begins at Ala20 just after the lipobox (residues 16–19). High, low, and negative ^1^H-^15^N-NOE values indicate low, medium, and high flexibility, respectively. (C) Sequence of LpoP starting at residue Ala20 just after the lipobox segment, colored as in (B).

To obtain structural information for LpoP, we used a hybrid approach involving both NMR and crystallography. This was required due to the predicted combination of both structured and intrinsically disordered regions in LpoP, which could not be probed optimally by either technique alone. LpoP is a predicted lipoprotein of 259 amino acids with a clear lipobox signal sequence that promotes localization and attachment to the *P. aeruginosa* outer membrane via an N-terminal lipid modification at Cys19 (Greene et al., 2018). The ^1^H-^15^N correlation spectrum of ^15^N-labeled LpoP (residues 20–259 with signal sequence removed for optimal solubility) displayed both dispersed peaks and intense narrow peaks with a very low ^1^H chemical shift dispersion, confirming the presence of structured and unstructured regions (Figure 1A). NMR data were recorded on a [^15^N-^13^C] LpoP sample and the backbone and side chain ^1^H-^15^N-^13^C resonances were assigned using conventional tri-dimensional experiments. The assigned spectra revealed that residues Asp 154 to Ser 258 form a globular domain consisting of a succession of six helices (as determined by chemical shift index analysis––Figure S1) (Berjanskii and Wishart, 2005). ^1^H-^15^N nuclear Overhauser effect (NOE) relaxation measurements displayed positive NOE values (>0.6) for residues 154–258, in agreement with the presence of a globular and stable domain (Figure 1A). With the exception of the short stretch from residues 147 to 153 with intermediate NOE values (0.15–0.4), the rest of the residues produced low or negative NOE values, which indicated fast motion (Figure 1B). Hence, the periplasmic part of LpoP comprises an N-terminal intrinsically disordered domain (residues 20–146) that is connected by a short segment with reduced mobility (residues 147–153) to the C-terminal globular domain (residues 154–258) (Figure 1C).

### Structure of the Globular C-Terminal Domain of LpoP

**Figure 2:**
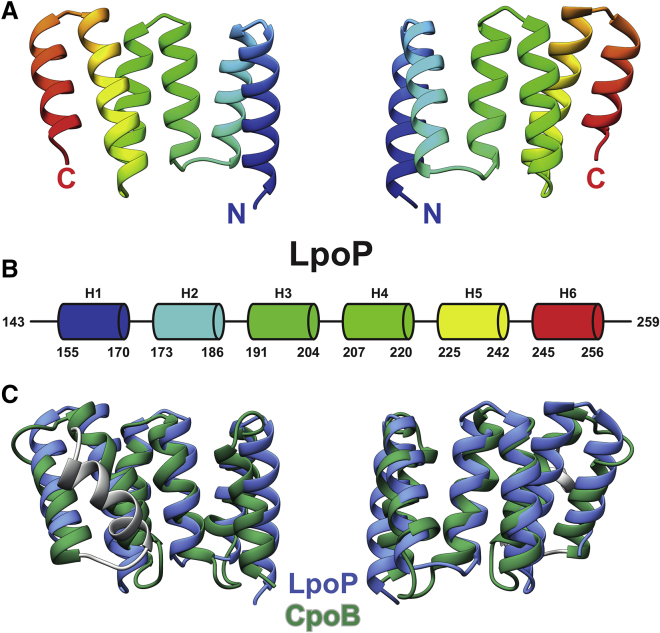
Structure of the Globular Domain of LpoP (A) Structure of LpoP_143-259_ determined by X-ray crystallography. (B) Schematic representation of the LpoP_143-259_ secondary structure. (C) Alignment of crystal structures of *P. aeruginosa* LpoP_143-259_ (blue) and *Xanthomonas campestris* CpoB_3-124_ (green and grey).

Crystals of *P. aeruginosa* LpoP_143-259_ displayed monoclinic P2_1_ symmetry with unit cell dimensions of a = 48.8 Å, b = 154.6 Å, c = 54.1 Å, and β = 90.1°, and diffracted with a resolution of 2.2 Å. There are eight molecules of LpoP in the asymmetric unit. The structure solution was phased using molecular replacement and a homology model of LpoP_143-259_ and final refinement statistics were generated (Table S1). The resulting maps showed well-resolved electron density for most of the protein chain, allowing near complete tracing of the ordered part from residues 152 onward (see the STAR Methods). The structure indicated a monomeric form of the protein with no obvious crystallographic formation of larger oligomers. LpoP_143-259_ is made up of six α helices (H1–H6) of variable length that form helix-turn-helix motifs similar to those of TPRs and that are linked together through short loops (Figures 2A and 2B) (Cortajarena et al., 2004, Zeytuni and Zarivach, 2012). Numerous inter-helical hydrophobic contacts favored by the high percentage of Leu (12%) and Ala (22%) stabilize the core structure (Figure S2A). LpoP has a striking structural similarity to protein domains formed by repetition of TPR domains (prosite PS50293 family). In particular, LpoP has a structure similar to the TPR domain of CpoB (PDB: 2XEV; Krachler et al., 2010) (root-mean-square deviation [RMSD] = 1.2 Å over main chain atoms from 59 residues and 2.5 Å over all 100 residues, in comparison with an RMSD of 0.3 Å over all 104 residues among the 8 LpoP molecules in the asymmetric unit) despite a limited sequence identity of 18% (Figure 2C) and varied electrostatic surface potential (Figure S2B). There was no significant structural conservation observed between LpoP and either of the activators LpoA (Vijayalakshmi et al., 2008) and LpoB (King et al., 2014) (Figure S2C).

### LpoP Interacts with ^*Pa*^PBP1B and Stimulates Its Activities *In Vitro*

**Figure 3:**
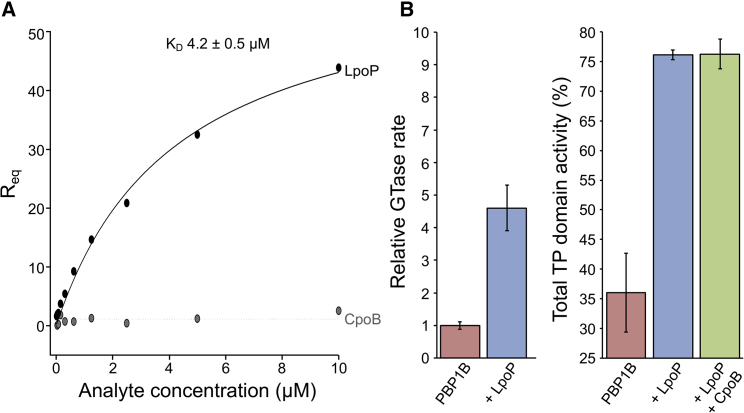
LpoP Interacts Directly with ^*Pa*^PBP1B to Activate the Synthase (A) Representative SPR binding curves resulting from LpoP or ^*Pa*^CpoB injection over immobilized ^*Pa*^PBP1B. The concentration of analyte protein injected is plotted against the response to its specific binding, or lack thereof, elicited at equilibrium. The protein injected is indicated next to the corresponding curve. Non-linear regression assuming one-site saturation was used to calculate the dissociation constant, K_D_. The K_D_ shown is the mean with standard deviation (SD) for n = 4. (B) Quantification of relative GTase reaction rate and TPase domain activity (the sum of both peptide crosslinking and carboxypeptidase activity) of ^*Pa*^PBP1B with and without LpoP and/or ^*Pa*^CpoB. The data shown are the mean with SD for n = 4 and 3, respectively. Corresponding representative high-performance liquid chromatography chromatograms and GTase data are shown in Figure S3.

LpoP was identified as a potential ^*Pa*^PBP1B activator based on the genetic observation that the presence of LpoP is required for the function of ^*Pa*^PBP1B in the cell (Greene et al., 2018). We wished to push this idea further to show that, like the *E. coli* LpoB/PBP1B activator/synthase pair, a direct physical interaction of LpoP with ^*Pa*^PBP1B is the underlying basis for activation. To test for this interaction *in vitro*, we conducted surface plasmon resonance (SPR) experiments with the purified proteins (Figure 3A). LpoP bound to immobilized ^*Pa*^PBP1B with a K_D_ of 4.2 ± 0.5 μM and approaching binding saturation at a concentration of approximately 10 μM. Monitoring the rate of GTase activity in the presence and absence of LpoP in a continuous assay (Egan and Vollmer, 2016) revealed that LpoP stimulated the GTase rate of ^*Pa*^PBP1B by 4.5-fold (Figures 3B and S3B). In addition, an endpoint TPase activity assay showed a 2-fold increase in transpeptidation products synthesized by ^*Pa*^PBP1B in the presence of LpoP (Figures 3B and S3C).

### ^*Pa*^CpoB Is Not a Regulator of ^*Pa*^PBP1B *In Vitro*

In an *in vitro* assay of ^*Pa*^PBP1B TPase activity, the addition of ^*Pa*^CpoB to a reaction containing ^*Pa*^PBP1B and LpoP did not alter crosslinking of the PG product (Figures 3B and S3C). This result was consistent with our SPR analysis showing that ^*Pa*^PBP1B did not interact with ^*Pa*^CpoB over the concentration range tested (Figure 3A). This is in contrast to the interaction of *E. coli* CpoB with PBP1B and its reduction of the stimulation of the TPase activity by LpoB.

### C-Terminal Globular Domain of LpoP Interacts with the UB2H Domain of ^*Pa*^PBP1B

**Figure 4:**
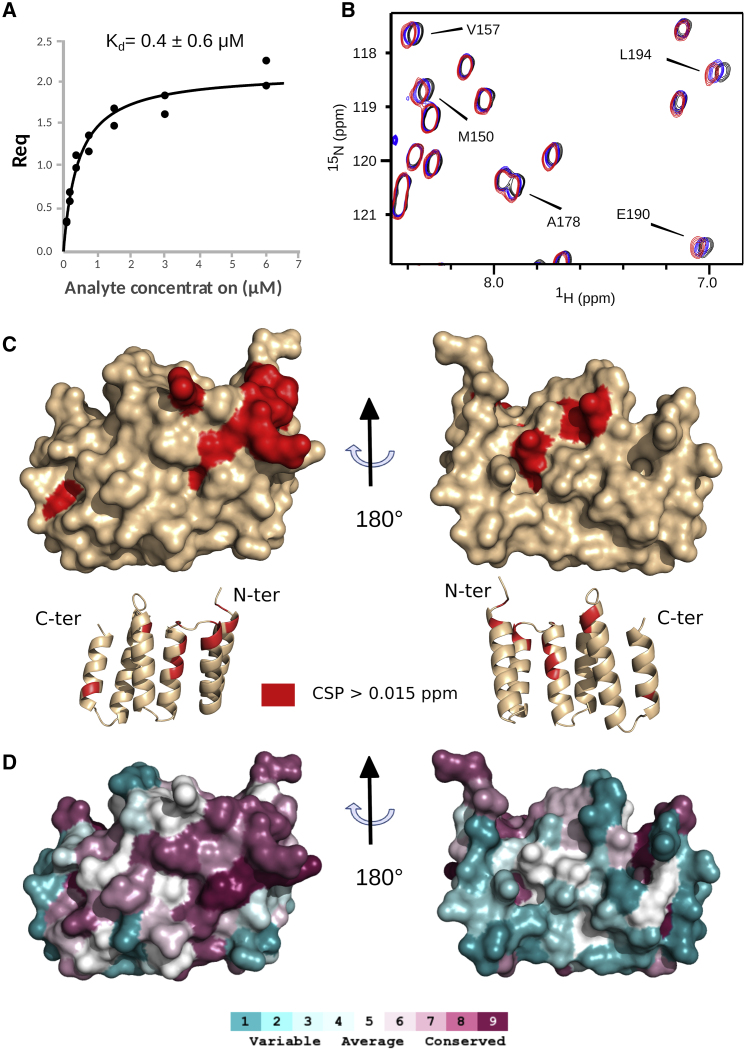
Interaction of LpoP with the ^*Pa*^UB2H Domain of ^*Pa*^PBP1B (A) Quantified BLI binding data for biotin-labeled ^*Pa*^UB2H binding to LpoP. (B) Region of ^1^H–^15^N correlation spectra showing chemical shift perturbations induced on ^15^N-labeled LpoP by addition of different ratios of ^*Pa*^UB2H. The spectra plotted in black, blue, and red correspond to a UB2H/LpoP ratio of 0, 1.2, and 1.8, respectively. The samples were prepared with 115 μM of ^15^N LpoP in 50 mM HEPES buffer (pH 7.0) containing 150 mM NaCl. ^*Pa*^UB2H was concentrated in the exact same buffer to reach a final concentration of 150 μM and added to the NMR tube to obtain the different ratios. The NMR experiments were recorded at 25°C and on a 20-T NMR spectrometer. (C) Residues showing a higher perturbation than 0.015 ppm upon UB2H addition are mapped in red on the surface and cartoon representation of the LpoP structure. (D) Sequence conservation scores were calculated using the ConSurf webserver (Landau et al., 2005) and displayed on the surface representation of LpoP using the same orientation as (C). Scores range from 1 (not conserved, cyan) to 9 (highly conserved, magenta).

In *E. coli*, LpoB interacts with the regulatory UB2H domain of PBP1B. Since a similar domain is present in ^*Pa*^PBPB1B (residues 66–159, 24% identity to *E. coli*), we used biolayer interferometry (BLI) to test for the interaction and quantify equilibrium dissociation constants (K_D_) of the ^*Pa*^UB2H-LpoP complex (Figure S4). The BLI curves were subjected to kinetic analysis and a K_D_ of 0.4 ± 0.6 μM was determined (Figure 4A). This K_D_ is slightly lower than the one obtained for the full ^*Pa*^PBP1B by SPR (4.2 ± 0.5 μM) but this difference could be due to the specific buffer required to measure the K_D_ by BLI using the truncated UB2H domain. However, the similarity between the two affinities suggests that the complex between LpoP and ^*Pa*^PBP1B is largely stabilized by a direct interaction of LpoP with the UB2H domain.

Superimposition of [^1^H,^15^N] BEST-TROSY spectra (Figure 4B) of LpoP in the presence or absence of ^PA^UB2H readily identified perturbed LpoP amide resonances, and the progressive shifts observed for the different concentration suggests a rapid exchange regime between the two domains, in agreement with the moderate affinity constant measured for this complex. Chemical shift perturbation was determined for each amino acid resonance and reported in a graph (Figure S5). The maximal chemical shift perturbations were mapped on the LpoP structure (Figure 4C) and found to concentrate mainly in the region connecting the extremities of helix 2 and helix 3. As shown in Figure 4D, this region also contains the most conserved amino acid residues as obtained by alignment of 150 sequences using ConSurf software.

### LpoP Likely Interacts between the GTase and UB2H Domains of ^*Pa*^PBP1B

**Figure 5:**
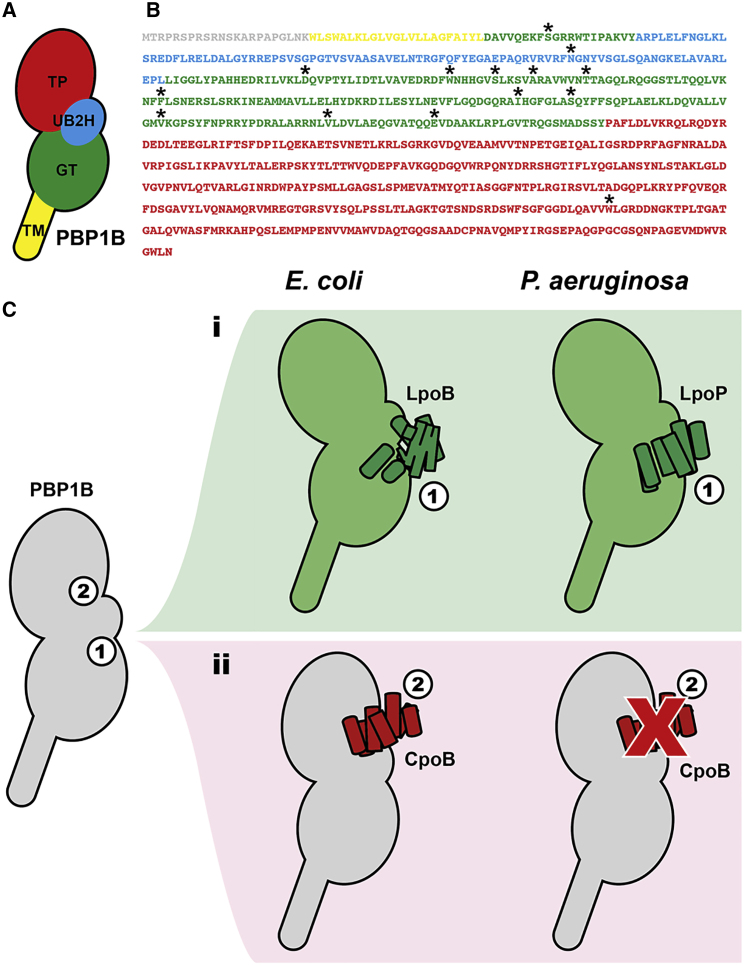
Interaction between LpoP and ^*Pa*^PBP1B (A) A scheme of the domains of ^*Pa*^PBP1B. (B) The sequence of ^*Pa*^PBP1B with potential interacting residues labeled with an asterisk. Asterisks denote low to medium confidence (>0.2) probability scores from GREMLIN co-evolutionary analysis between LpoP and ^*Pa*^PBP1B. (C) A schematic representation of the proposed model for ^*Pa*^PBP1b activation and regulation, in comparison with ^*Ec*^PBP1b. We propose a folding-independent activator binding site between the UB2H and GTase domains in ^*Pa*^PBP1B (i) and the absence of an interaction between ^*Pa*^CpoB and ^*Pa*^PBP1B in *P. aeruginosa* (ii).

We have been unable to produce a stable ^15^N-labeled UB2H domain, which prevented us from performing further experiments on the interface residues in ^*Pa*^PBP1B. Therefore, we turned to co-evolutionary analysis of ^*Pa*^PBP1B and LpoP (GREMLIN; Ovchinnikov et al., 2014). While LpoP is not widely conserved among Gram-negative bacteria, there was a small subset of species with both a PBP1B and LpoP homolog (Greene et al., 2018), resulting in putative (with medium to low confidence) interface residues in ^*Pa*^PBP1B (Figures 5A and 5B). These data suggest that LpoP likely also associates with the GTase domain of ^*Pa*^PBP1B, sitting between the UB2H and GTase domains, as has been proposed for the binding site for *E. coli* LpoB with ^*Ec*^PBP1B.

## Discussion

The recent identification of LpoP, which is required for the proper function of PBP1B in *Pseudomonas* (Greene et al., 2018), prompted this biochemical and biophysical analysis of LpoP. In this work, we performed an in-depth investigation via NMR spectroscopy, biochemical assays to probe activity and binding, X-ray crystallography, and co-evolutionary analysis. We reveal the semi-conserved nature of class A PBP activation, highlighting the similarities and differences between LpoB- and LpoP-based systems.

### Commonalities between LpoB- and LpoP-Based Systems

We show that the domain organization of LpoP is consistent with the model that it serves to activate ^*Pa*^PBP1B in an analogous manner to that by which LpoB activates ^*Ec*^PBP1B. Using NMR, we show that LpoP has an intrinsically disordered N-terminal extension, followed by a structured globular domain that specifically interacts with the UB2H domain of ^*Pa*^PBP1B. This is similar to the previously found architecture of LpoB, which has been proposed to activate ^*Ec*^PBP1B in response to the porosity of the PG layer (Typas et al., 2010, Typas et al., 2012). According to this model, LpoB activates the synthase at sites with stretched PG. Recent work showed that ^*Ec*^PBP1B/LpoB also forms a PG repair complex with the LD-transpeptidase LdtD and the DD-carboxypeptidase PBP6A, which was hypothesized to repair faults in PG that arise upon severe outer membrane assembly defects (Morè et al., 2019). Based on the similar domain organization of LpoB and LpoP and the observed activation of ^*Pa*^PBP1B, we hypothesize that LpoP has analogous roles in the *P. aeruginosa* pathogen.

We propose that LpoP would not only act to regulate ^*Pa*^PBP1B at this broad mechanistic level, but also in a similar way at the protein level. We observed that LpoP interacts strongly with the UB2H domain via SPR analysis, and our co-evolutionary analysis convincingly suggests that this interaction interface is roughly spatially conserved with that of ^*Ec*^PBP1B-LpoB (Figure 5). Together with previous data showing that it is possible to generate hyperactive PBP1B mutants that do not require activation by LpoB or LpoP in both species (Greene et al., 2018), this supports the notion for a common mechanism for Lpo binding to conserved regions between the UB2H and GTase domains, leading to similar conformational rearrangements (Figure 5B). These conformational rearrangements activate PBP1B in *E. coli* (Egan et al., 2014, Egan et al., 2018, Lupoli et al., 2014, Paradis-Bleau et al., 2010, Typas et al., 2010), and, likewise, we see a similar activation in the GTase and TPase activity of ^*Pa*^PBP1B by LpoP, further reinforcing a conserved pathway of conformational activation.

### Differences between LpoB- and LpoP-Based Systems

Despite the overarching conserved features between LpoB- and LpoP-based PBP1B activation mechanisms described above, there are also key differences. First, the structure of the C-terminal globular domain of LpoP is distinct from that of LpoB. LpoP's C-terminal globular domain exclusively consists of three repeated TPR motifs, giving it primarily alpha-helical character and a structure greatly similar to the N-terminal domain of CpoB (Figures 2C and S2). This is in stark contrast to the C-terminal domain of LpoB, which consists of an internal 4-strand β sheet flanked by N- and C-terminal helices with an overall structural similarity to the N-terminal domain of TolB (Egan et al., 2014, King et al., 2014) (Figure S2C).

It appears that, despite the stark difference between the globular domains of LpoB and LpoP, the use of TPR motifs in the regulation of class A PBP proteins is a common theme. We see the use of TPR motifs in both the negative regulator CpoB, as well as in the LpoA activator of *E. coli* PBP1A. Interestingly, in the case of LpoA, these TPR repeats are seen to be involved in the extension of Lpo across the periplasm, instead of a direct interaction module as seen in LpoP and CpoB. Regardless, it is remarkable how activators and regulators of class A PBPs evolved from similar domain pieces in these distinct lineages.

Beyond the purely structural differences between the LpoP and LpoB systems, the different effects of CpoB on the two activators is of interest. Here, we see that ^*Pa*^CpoB does not reduce the activation of the TPase activity of ^*Pa*^PBP1B by LpoP. This is explained by the lack of an interaction between ^*Pa*^CpoB and ^*Pa*^PBP1B. In *E. coli* the *cpoB* gene is adjacent to the genes for the Tol-Pal apparatus and CpoB has been proposed to act as a link between outer membrane constriction and PG synthesis activity. Perhaps CpoB primarily plays a role in the Tol-Pal apparatus, and interaction with PG synthases evolved downstream in a subset of Gammaproteobacteria. It will be of interest to further test this hypothesis by determining potential alternate roles for CpoB in *Pseudomonas* strains, and if the potential lack of a PG synthase regulatory function correlates to the presence of an LpoP.

### Conclusion

We report the structure and organization of LpoP and its interaction site with PBP1B from *P. aeruginosa*. We highlight the similarities and key differences between this activator-PG synthase system and the LpoB-based PBP1B activation in *E. coli*. Our data expand upon a semi-conserved mechanism for PBP1B activation in *P. aeruginosa* and other LpoP-based systems first proposed by Greene et al. (2018). Using this knowledge of the specificities of LpoP- and LpoB-based class A PBP regulation, we hope to inform the future study and potential inhibition of relevant pseudomonal pathogens. Nosocomial infections from the latter are a major health burden to immunocompromised, AIDS, burn, and cystic fibrosis patients, for example, and a species-directed antimicrobial to lessen the long-term respiratory burden inflicted by these strains is direly needed.

## STAR★Methods

### Key Resources Table

**Table:** 

REAGENT or RESOURCE	SOURCE	IDENTIFIER
**Bacterial and Virus Strains**		

BL21	NEB	C2527I

**Chemicals, Peptides, and Recombinant Proteins**		

Thrombin	Novagen	69671-3
cOmpleteTM EDTA-free protease inhibitor	Roche	11873580001
Ni-NTA	Qiagen	30210
Ni-NTA Superflow	Qiagen	30410
Bocillin	ThermoFisher	B13233

**Deposited Data**		

CpoB	(Krachler et al., 2010)	PDB ID: 2XEV
BcsC	(Nojima et al., 2017)	PDB ID: 5XW7
LpoA	(Vijayalakshmi et al., 2008)	PDB ID: 3CKM
LpoB	(King et al., 2014)	PDB ID: 4Q6Z
LpoP	this study	PDB ID: 6W5Q

**Oligonucleotides**		

pET28 linearization forward primer: ATGGCTAGCATGACTGGTGGAC	this study	n/a
pET28 linearization reverse primer: ATGGCTGCCGCGCGGCACCAG	this study	n/a
^*Pa*^PBP1B SLIC forward primer: TGGTGCCGCGCGGCAGCCATATGAC GCGTCCCCGATCCC	this study	n/a
^*Pa*^PBP1B SLIC reverse primer: TGTCCACCAGTCATGCTAGCCATTTA TTCAATTCAGCCAGCCACGTAC	this study	n/a
LpoP SLIC forward primer: CTGGTGCCGCGCGGCAGCCATGC CAGCCCGCAGCACGGGG	this study	n/a
LpoP SLIC reverse primer: TGTC- CACCAGTCATGCTAGCCATTATCAG GAGCTGACCTTGGCCT	this study	n/a
^*Pa*^CpoB SLIC forward primer: CTGGTGCCGCGCGGCAGCCAT ATGCCCAAGCACCTGCGTGT	this study	n/a
^*Pa*^CpoB SLIC reverse primer: TG TCCACCAGTCATGCTAGCCATT TAGCGAAGGTTCTTGAGATCGCGC	this study	n/a

**Recombinant DNA**		

pET28a	Novagen	69864-3
^*Pa*^PBP1B Expression	this study	pAJFE52
LpoP Expression	this study	pAJFE57
LpoP_142-259_Expression	this study	NC_50
^*Pa*^UB2H Expression	this study	UB2H_exp
^*Pa*^CpoB Expression	this study	pAJFE50

**Software and Algorithms**		

SigmaPlot 13	Systat Software Inc.	n/a
ForteBio Data Analysis Software	ForteBio	n/a
XDS	(Kabsch, 2010)	n/a
Rosetta	(Leaver-Fay et al., 2011)	n/a
AMPLE	(Bibby et al., 2012)	n/a
SWISS-MODEL	(Waterhouse et al., 2018)	n/a
Buccaneer	(Cowtan, 2006)	n/a
Phenix	(Adams et al., 2010)	n/a
Coot	(Emsley and Cowtan, 2004)	n/a
TopSpin™	Bruker	n/a
CcpNmr Analysis Software	(Vranken et al., 2005)	n/a
GREMLIN	(Ovchinnikov et al., 2014)	n/a

### Lead Contact and Materials Availability

Further information and requests for resources should be directed to and will be fulfilled by the Lead Contact, Dr. Jean-Pierre Simorre, e-mail: jean-pierre.simorre@ibs.fr). This study did not generate new unique reagents.

### Experimental Model and Subject Details

Proteins were recombinantly expressed in the *E.coli* BL21 (DE3) strain carrying plasmids listed in the Key Resources Table.

### Method Details

#### Plasmid Construction

Expression vectors for ^*Pa*^PBP1B (pAJFE52), LpoP lacking its lipoprotein sorting sequence (residues 20-259) (pAJFE57), and ^*Pa*^CpoB lacking its periplasmic export sequence (residues 22-274) (pAJFE50) have been prepared by Sequence and Ligase Independent Cloning (SLIC) following the procedure described previously (Jeong et al., 2012). Genes have been inserted into pET28a, which had been linearized and amplified by PCR, opening the vector at the NdeI restriction site. Resulting proteins possessed an N-terminal hexa-Histidine tag followed by a thrombin cleavage sequence. pET28 linearization forward primer: ATGGCTAGCATGACTGGTGGAC. pET28 linearization reverse primer: ATGGCTGCCGCGCGGCACCAG. Gene inserts, with SLIC compatible complementary overhang sequences for insertion into the linearized pET28, have been amplified from *P. aeruginosa* PA01 genomic DNA template. ^*Pa*^PBP1B SLIC forward primer: TGGTGCCGCGCGGCAGCCATATGACGCGTCCCCGATCCC. ^*Pa*^PBP1B SLIC reverse primer: TGTCCACCAGTCATGCTAGCCATTTATTCAATTCAGCCAGCCACGTAC. LpoP SLIC forward primer: CTGGTGCCGCGCGGCAGCCATGCCAGCCCGCAGCACGGGG. LpoP SLIC reverse primer: TGTC- CACCAGTCATGCTAGCCATTATCAGGAGCTGACCTTGGCCT. ^*Pa*^CpoB SLIC forward primer: CTGGTGCCGCGCGGCAGCCATATGCCCAAGCACCTGCGTGT. ^*Pa*^CpoB SLIC reverse primer: TGTCCACCAGTCATGCTAGCCATTTAGCGAAGGTTCTTGAGATCGCGC.

#### LpoP Protein Purification

For LpoP purification, pAJFE57 was transformed into BL21(DE3). This strain was cultured in 1.5 L of LB at 30°C to an OD578 of 0.5, at which point 1 mM IPTG was added to induce protein overproduction for 3 h at 30°C. The culture was rapidly cooled before harvesting by centrifugation, cells have been resuspended in 80 mL 25 mM Tris/HCl, 500 mM NaCl, 20 mM imidazole, 10% glycerol, pH 7.5. Sigma protease inhibitor cocktail (PIC) at 1 in 1000 dilution and 100 μM PMSF have been added to the resuspension before cells have been disrupted by sonication (Branson digital sonifier). Lysed cells have been fractionated by ultracentrifugation (130,000 x *g*, 1 h, 4°C). The soluble fraction was applied to a 5 mL HisTrap column attached to an ÄKTA Prime+ equilibrated in resuspension buffer indicated above. The column was washed with 25 mM Tris/HCl, 1 M NaCl, 40 mM imidazole, 10% glycerol, pH 7.5 before elution of bound protein with 25 mM Tris/HCl, 500 mM NaCl, 400 mM imidazole, 10% glycerol, pH 7.5. His-LpoP containing fractions have been pooled and 4 U/mL restriction grade thrombin (Novagen) was added. The sample was dialysed against 20 mM Tris/HCl, 200 mM NaCl, 10% glycerol, pH 7.5 for 20 h at 4°C. The sample was applied to a size exclusion chromatography column (HiLoad 16/600 Superdex200 pg) equilibrated in 20 mM HEPES/NaOH, 200 mM NaCl, 10% glycerol, pH 7.5 attached to an ÄKTA Prime+ system. The sample was resolved at 0.8 mL/min. Fractions have been collected and concentrated to 4 mL.

#### Labeled LpoP for NMR Spectroscopy

For LpoP purification, pAJFE57 was transformed into BL21(DE3). This strain was cultured in 1 L of M9 containing 1g/L of ammonium chloride and 2g/L of glucose at 37°C to an OD600nm of 0.6, at which point 1 mM IPTG was added to induce protein overproduction for 4 h at 30°C. After harvesting, cells have been resuspended in 15 mL 20 mM Tris pH8, 300 mM NaCl buffer supplemented with 1 tablet of cOmpleteTM EDTA-free (Roche) as protease inhibitor cocktail. Cells have been disrupted by sonication (Sonics Vibra CellTM). Lysed cells have been clarified by centrifugation (46 000 g, 40 min, 4°C). The soluble fraction was applied to a 4 mL Ni-NTA (Qiagen) column beforehand equilibrated in resuspension buffer. After sample application the column was washed with equilibration buffer containing 25 mM imidazole. Elution was performed using the same buffer supplemented with 500 mM imidazole. Fractions containing the protein have been pooled and injected to a size exclusion chromatography column (HiLoad 26/600 Superdex75 pg) equilibrated in 20 mM Tris pH8, 300 mM NaCl buffer. Fractions have been dialysed against 50 mM HEPES pH 7, 150 mM NaCl buffer for NMR or dialysed against 20 mM Tris, 150 mM NaCl, pH 8 for BLI interactions tests.

#### ^LpoP^_142-259_ Protein Purification

Plasmid expressing LpoP_142-259_ (NC_50) has been transformed into *E. coli* BL21(DE3) expression strain. This strain has been cultured in LB at 37°C to an OD600 of 0.6, at which point 1 mM IPTG has been added to induce protein overproduction overnight at 25°C. For purification of LpoP_142-259_, cell pellets have been resuspended in lysis buffer (20 mM HEPES, pH 8.0, 300 mM NaCl, 10% glycerol) and lysed by processing twice with a homogenizer (15 kPa; Avestin). Cellular debris was pelleted by centrifugation at 125,000 x *g* for 1 hour. The resultant supernatant was loaded onto 10 mL Ni2+-saturated Ni-NTA superflow beads (Qiagen), washed with 65 mM imidazole in 20 mM HEPES, pH 8.0, 300 mM NaCl, and the protein was eluted with 300 mM imidazole in the previous buffer. 1 U of thrombin was added per mg of protein to remove the N-terminal His-tag overnight at 4°C). Samples have been purified further by size exclusion chromatography (SEC) with a Superdex 200 column (GE Lifesciences) equilibrated in 20 mM HEPES, pH 8.0, 150 mM NaCl. Fractions containing pure LpoP have been pooled and concentrated to 30 mg/mL. Protein was frozen rapidly in liquid nitrogen and stored at -80°C until required.

#### ^Pa^PBP1B Protein Purification

^*Pa*^PBP1B was prepared by largely the same procedure as its *E. coli* homologue, described previously (Bertsche et al., 2006) with modifications. Plasmid pAJFE52 was transformed into *E. coli* BL21(DE3) expression strain. This strain was cultured in 1.5 L of LB at 30°C to an OD578 of 0.5, at which point 1 mM IPTG was added to induce protein overproduction for 3 h at 30°C. The culture was rapidly cooled before harvesting by centrifugation, cells have been resuspended in 80 mL 25 mM Tris/HCl, 500 mM NaCl, 1 mM EGTA, 10% glycerol, pH 7.5. Sigma protease inhibitor cocktail (PIC) at 1 in 1000 dilution and 100 μM PMSF have been added to the resuspension before cells have been disrupted by sonication (Branson digital sonifier). Lysed cells have been fractionated by ultracentrifugation (130,000 x *g*, 1 h, 4C). Insoluble material was resuspended in 45 mL 25 mM Tris/HCl, 5 mM MgCl_2_, 1 M NaCl, 20% glycerol, 2% Triton X-100, pH 7.5 plus 1/1000 PIC and 100 M phenylmethylsulphonylfluoride (PMSF) and mixed overnight at 4°C. Remaining insoluble material was pelleted by a second ultracentrifugation step, leaving solubilised membrane protein in the supernatant. The sample was diluted 1:1 with 25 mM Tris/HCl, 1 M NaCl, 40 mM imidazole, 20% glycerol, pH 7.5 before application to a 5 mL HisTrap column attached to an ÄKTA Prime+ equilibrated in 25 mM Tris/HCl, 2.5 mM MgCl_2_, 1 M NaCl, 20 mM imidazole, 20% glycerol, 1% Triton X-100, pH 7.5. After binding the column was washed with 25 mM Tris/HCl, 1 M NaCl, 50 mM imidazole, 20% glycerol, 0.2% Triton X-100, pH 7.5 before elution of bound protein with 25 mM Tris/HCl, 1 M NaCl, 400 mM imidazole, 20% glycerol, 0.2% Triton X-100, pH 7.5. His-^*Pa*^PBP1B containing fractions have been pooled and 4 U/mL restriction grade thrombin (Novagen) was added. The sample was dialysed against 25 mM Tris/HCl, 1 M NaCl, 0.5 mM EGTA, 10% glycerol, pH 7.5 for 20 h at 4°C. The sample was then dialysed against a sequence of buffers in preparation for ion exchange chromatography. Firstly 4 h against 20 mM NaAc, 1 M NaCl, 10% glycerol, pH 5.0, followed by 16 h against 20 mM NaAc, 300 mM NaCl, 10% glycerol, pH 5.0. The sample was diluted 1:1 with 20 mM NaAc, 50 mM NaCl, 10% glycerol, 0.2% reduced Triton X-100, pH 5.0 before application to an equilibrated 1 mL HiTrap SP column attached to an ÄKTA Prime+ system. The column was equilibrated in buffer A (20 mM NaAc, 200 mM NaCl, 10% glycerol, 0.2% reduced Triton X-100, pH 5.0). ^*Pa*^PBP1B was eluted by gradient from 100% buffer A to 100% buffer B (20 mM NaAc, 2 M NaCl, 10% glycerol, 0.2% reduced Triton X-100, pH 5.0) over 14 mL. ^*Pa*^PBP1B containing fractions have been pooled and dialysed against 20 mM NaAc, 500 mM NaCl, 20% glycerol, 0.2% reduced Triton X-100, pH 5.0. To ensure correct folding, the protein’s ability to bind to the fluorescent β-lactam Bocillin was assayed as previously described (Egan et al., 2018) (Figure S3). ). Briefly, Bocillin binding was assessed by incubating 1 μg of protein in 20 μL of 20 mM HEPES/NaOH pH 7.5, 5 mM MgCl_2_, 150 mM NaCl, 0.05% Triton X-100 with or without 1 mM ampicillin at 37°C for 30 min. After which 50 ng of BOCILLINTM FL (Bocillin; Invitrogen, USA) was added before further incubation at 37°C for 30 min. 15 μL of each sample was resolved by SDS-PAGE, the gel then imaged by Typhoon scanner 9400 with excitation and emission filters of 488 and 520 nm, respectively. The same gel was then stained using Coomassie Brilliant-Blue.

#### ^Pa^UB2H Domain Purification

Plasmid carrying UB2H domain of *Pseudomonas aeruginosa* PBP1b (UB2H_exp) was transformed in Bl21(DE3) competent cells. This strain was cultured to an OD_600nm_ of 0.7, at which point 1 mM IPTG was added to induce protein overproduction for 3h at 37°C. After harvesting cells, pellet was resuspended in 20 mL 50 mM Tris pH 7.5 buffer. Cells have been disrupted by sonication (Sonics Vibra CellTM) and lysate was centrifuged in a Beckman cold centrifuge (30 minutes, 46000g). The supernatant was discarded and inclusion bodies containing ^*Pa*^UB2H was washed following cellular fractionation protocol alternating four washing steps in different buffer and centrifugations. The centrifugation steps have been done at 4°C during 30 min at 46000g. Washing steps have been done using 20 mL of 50 mM Tris pH 7.5, 2 M NaCl buffer, then 50 mM Tris pH 7.5, 1% Triton X100 buffer, finally two washes of 20mL 50 mM Tris pH7.5 . At the end inclusion bodies have been solubilized in 80mL of 50 mM Tris pH 7.5, 6M Guanidium during an overnight incubation. After centrifugation (30 min at 46 000g, 4°C) the supernatant was loaded on 4 mL of Ni-NTA (Qiagen) column. Column was washed with 50mM Tris pH 7.5, 6M guanidium, 25 mM Imidazole buffer, and the protein was eluted with 50mM Tris pH 7.5, 6M guanidium, 500mM imidazole. The protein was refolded performing three baths of dialysis against 100mM sodium acetate pH5 buffer. After centrifugation the soluble protein was injected to a size exclusion chromatography column (HiLoad 26/600 Superdex75 pg) equilibrated in 100mM sodium acetate pH 5 buffer. ^*Pa*^UB2H was eluted in a single peak corresponding to a monomer of the protein.

#### ^Pa^CpoB Protein Purification

For ^*Pa*^CpoB purification; protein overproduction, immobilised metal affinity chromatography (IMAC), and size exclusion chromatography (SEC) have been performed by the same procedure as for LpoP described above. An additional ion exchange chromatography step, adapted from (Krachler et al., 2010), was included between IMAC and SEC. Post IMAC, His-^*Pa*^CpoB containing fractions have been pooled, 4 U/mL thrombin added, and dialysed against 20 mM Tris/HCl pH 8.0 for 20 h at 4°C. Some impurities carried from IMAC precipitate during this stage, these have been removed after dialysis by centrifugation (4000 g, 15 min, 4C). The sample was applied to a 5 mL HiTrap SP column attached to an ÄKTA Prime+, equilibrated in buffer A (20 mM Tris/HCl pH 8.0). Bound ^*Pa*^CpoB was eluted by gradient from 100% buffer A to 100% buffer B (20 mM Tris/HCl, 500 mM NaCl, pH 8.0) over 50 mL. In SEC, ^*Pa*^CpoB eluted as a single peak, consistent with a trimer, as reported for its *E. coli* homologue (Krachler et al., 2010).

#### *In vitro* Protein Interaction and Activity Assays

Surface plasmon resonance (SPR) experiments have been performed as previously described (Egan et al., 2014) using a BioRad ProteOn XPR36 system and associated software (BioRad) with a GLC amine coupling sensorchip. LpoP and PaCpoB samples have been prepared for injection over the PaPBP1B surface by 1:1 serial dilution from 10 μM to 19.5 nM. Assays have been performed at 25°C, at a flow rate of 75 μL/min and with an injection time of 5 min. The running buffer consisted of 10 mM Tris/HCl, 150 mM NaCl, 0.05% Triton X-100, pH 7.5. The dissociation constant (Kd) was calculated by non-linear regression using SigmaPlot 13 software (Systat Software Inc.). Continuous fluorescence GTase assays have been performed as described previously (Egan and Vollmer, 2016) with slight modification. ^Pa^PBP1B was assayed at a concentration of 0.5 μM 10 μM LpoP at 37°C for 1 h. Time points have been taken every 1 min, instead of every 20 s to reduce photobleaching. Measurement of total PG synthesis activity using radiolabelled lipid II substrate was also performed as previously described (Biboy et al., 2013) using 0.5 M enzyme with 10 μM LpoP, 50 μM PaCpoB at 37°C for 3 h. Total TPase activity was calculated as the percentage of muropeptide products known to be produced by this domain’s function, including peptide cross-linking and DD-carboxypeptidase activity.

#### Biolayer Interferometry (Bli) Experiments

Biolayer Interferometry Experiments (BLI) have been recorded on an OctetRED96e (Fortebio) using biotinylated protein attached on streptavidin tips. For biotinylation of LpoP and ^*Pa*^UB2H, 100μL of protein at 3.3 mg/mL an 5,2 mg/mL respectively for LpoP and ^*Pa*^UB2H are mixed with 10μL of 1M MES pH 5,5, 2,7μL of Biotin-Hydrasin and 6,7μL of EDC (N-(3-Dimethylaminopropyl)-N′-ethylcarbodiimide). The reaction of biotinylation is performed 2h at 22°C under agitation. After biotinylation UB2H was dialysed in 50mM MES pH 6.5 and LpoP was dialysed in HBS buffer. Non-biotinylated LpoP and UB2H have been prepared in 50mM MES pH6.5 buffer at 1,88 mg/mL and 5,37mg /mL respectively. UB2H-Biot (25μg/ml) was immobilised on Streptavidin coated -BLI biosensors to reach about 1 nm immobilisation in HBS-T (10 mM Hepes pH 7,5,150 mM NaCl and 0.02% Tween) Buffer. Tips loaded with UB2H have been inserted into different LpoP concentrations (0 to 6μM) in HBS-T Buffer at 23°C. Kinetics have been recorded with 1200s for association and 1000sec for dissociation phases, experiments have been reproduced twice with 10mM HCl pulses (3x) used for regeneration between cycles. Responses at equilibrium of the two series (end of the association phase) have been simultaneously fitted with the equation $Req=\frac{Rmax\times conc}{Kd\times conc}$ by ForteBio data analysis software.

#### X-ray Crystallography and Structure Determination

*P. aeruginosa* LpoP protein was crystallized at 20°C by sitting drop vapour diffusion using 0.2 μL protein solution (30 mg/mL purified protein in 20 mM HEPES, pH 8.0, 150 mM NaCl) and 1 μL of mother liquor (0.1 HEPES pH 7.5, 2.5 M AmSO_4_, 1% PEG 400, 2.5% 1,2-butanediol). X-ray diffraction data of LpoP was collected on Advanced Light Source beamline 5.0.2. Diffraction data have been processed using XDS (Kabsch, 2010) and the structure was solved by molecular replacement using an ensemble of truncated Rosetta (Leaver-Fay et al., 2011) refined homology models in AMPLE (Bibby et al., 2012) based on a model from SWISS-MODEL (Waterhouse et al., 2018) and PDBID:5XW7 (Nojima et al., 2017). The structure was auto-built using Buccaneer (Cowtan, 2006) and subsequently refined using Phenix (Adams et al., 2010) and Coot (Emsley and Cowtan, 2004). See Table S1 for data collection and refinement statistics.

#### NMR Resonance Assignments

The 2D- and 3D-NMR experiments have been collected on 200 μM ^13^C,^15^N-labeled LpoP NMR samples in 30 mM HEPES, 200 mM KCl buffer at pH 7.5 containing 10%D_2_O. Backbone resonance assignments have been carried out using a combination of 2D ^1^H-^15^N-BEST-TROSY and 3D HN(CO)CACB, iHNCACB, HNCO, HN(CA)CO, H(NCACO)NH. In order to limit the number of overlaps, BEST-TROSY version of the above listed experiments was used (Brutscher et al., 2015, Solyom et al., 2013). For the assignment of side-chains aliphatic carbons, 2D ^1^H-^13^C-HSQC and 3D (H)C(CO)NH, (H)CCH-TOCSY, and H(C)CH-TOCSY experiments have been collected. All spectra have been recorded at 25°C using Bruker AVANCE spectrometers operating at 800 and 850 MHz proton frequency equipped with TCI cryoprobes.

The NMR spectra have been processed using the TopSpin™ software by Bruker in its 3.2 version and have been analyzed using the CcpNmr Analysis software (Vranken et al., 2005). The ^1^H chemical shifts have been referenced to the internal standard 4,4-dimethyl-4-silapentane-1- sulfonic acid (DSS) methyl resonance. ^13^C and ^15^N chemical shifts have been referenced indirectly using the IUPAC-IUB protocol (Markley et al., 1998).

#### NMR Titration Experiments

Interaction studies have been performed with ^13^C,^15^N-labeled LpoP at 115 μM prepared in a buffer containing 50 mM HEPES buffer, pH 7, 150 mM NaCl and 5% (vol/vol) D_2_O. Unlabelled ^*Pa*^UB2H have been dialyzed in the same buffer at a concentration of 150 μM and successively added to the NMR tube to reach the protein-to-protein ratio of 0, 0.6, 1.2 and 1.8. [^1^H,^15^N]-BEST-TROSY-HSQC spectra have been collected at 298K for each protein ratio using Bruker AVANCE spectrometers equipped with a TCI cryoprobe and operating at 850 MHz proton frequency.

#### Co-evolutionary Analysis

The Baker lab’s GREMLIN software (Ovchinnikov et al., 2014) was used to probe the interface between LpoP and ^*Pa*^PBP1b. Input sequences were that of the globular domain of LpoP and all residues of ^*Pa*^PBP1b. E-value cut-offs for both sequences have been set at 10^-2^ and the alignments have been run for 8 iterations.

#### Chemical Shift Perturbation Analysis

Analysis software CcpNmr 2.2 was used to monitor protein chemical shift perturbations for every assigned amide resonance by superimposition of the ^15^N-BEST-TROSY spectra and automatic peak picking. Chemical shift perturbations (Δδ) have been calculated on a per-residue basis for the highest substrate-to-protein ratio as described previously (Egan et al., 2018).

### Data and Code Availability

Atomic coordinates for the LpoP model have been deposited in the protein data bank with accession code 6W5Q [https://doi.org/10.2210/pdb6W5Q/pdb].

## Supplemental Information

Document S1. Figures S1–S5 and Tables S1

Document S2. Article plus Supplemental Information
