# Supplementary material for: Structure of the Peptidoglycan Synthase Activator LpoP in Pseudomonas aeruginosa
Source: Structure. 2020 Jun 2;28(6):643–650.e5. doi: 10.1016/j.str.2020.03.012 (PMC7267771; doi:10.1016/j.str.2020.03.012)
Supplement: Document S1. Figures S1–S5 and Tables S1 [file mmc1.pdf]

Structure, Volume *28*

Supplemental Information

Structure of the Peptidoglycan Synthase Activator LpoP in *Pseudomonas aeruginosa*

Nathanael A. Caveney, Alexander J.F. Egan, Isabel Ayala, Cédric Laguri, Craig S. Robb, Eefjan Breukink, Waldemar Vollmer, Natalie C.J. Strynadka, and Jean-Pierre Simorre

SUPPLEMENTAL INFORMATION

Structure of the peptidoglycan synthase activator LpoP in *Pseudomonas aeruginosa*

Nathanael A. Caveney[1,#], Alexander J. F. Egan[2,#], Isabel Ayala[3], Cédric Laguri[3], Craig S. Robb[1], Eefjan Breukink[4], Waldemar Vollmer[2,*], Natalie C. J. Strynadka[1,*], and Jean-Pierre Simorre[3,*]

[1]Department of Biochemistry and Molecular Biology and Centre for Blood Research, The University of British Columbia, Vancouver V6T 1Z3, British Columbia, Canada
[2]Centre for Bacterial Cell Biology, Biosciences Institute, Newcastle University, Richardson Road, Newcastle upon Tyne, NE2 4AX, United Kingdom
[3]Univ. Grenoble Alpes, CNRS, CEA, IBS, F-38000 Grenoble, France
[4]Department of Membrane Biochemistry and Biophysics, Utrecht University, Utrecht 3584 CH, The Netherlands.

[#]Authors contributed equally to the work

*corresponding authors ncjs@mail.ubc.ca, waldemar.vollmer@newcastle.ac.uk, jean-pierre.simorre@ibs.fr [1]

Lead contact : jean-pierre.simorre@ibs.fr

```
4            13        23        33        43        53
4   MASMTGGQQMGRGSEFASPQHGAIPVVDSGTPVSNQESGGFRITRTQVPR 53
4   CTTTTTTTTCCCCCCCCTTTTCCTTTTCCCCCCCCCCCCTTTBBCCCTTT 53
4                                                      53

54           63        73        83        93        103
54  TQQGAATQGIPQGGDSGVVVMVPQGANSAPIQTFPAQSGAAPISSAPLGT 103
54  TCCTTTTCCCCCCCTTTTBBBTTTTCCCCCCCCCCCCCCCCCCCCCCCCC 103
54                                                     103

104          113       123       133       143       153
104 GTQYQAPPSSASTPPLGGSYNMPPSGASRSAPTGIPASGSAGSLAADEQL 153
104 CCCCCCCCCCCCCTTTTCCCTTTTTTTTCCTTTTCCCCCCCCCCCCCCCC 153
104                                                    153

154          163       173       183       193       203
154 DGPVLAMLTTAQQQQGSGDLNSAAASLERAQRIAPREPQVLYRLAQVRLA 203
154 CHHHHHHHHHHHHHHHCCCHHHHHHHHHHHHHHHTTTTHHHHHHHHHHHH 203
154                                                    203

204          213       223       233       243       253
204 QGDAAQAEQVARRGLSYANGRPALQAGLWELIAQAREKQGDSAGAALARQ 253
204 CCCHHHHHHHHHHHHHHCCCCHHHHHHHHHHHHHHHHHHCCCHHHHHHHH 253
204                                                    253

254        259
254 KAKVSS 259
254 HCCCCC 259
254        259
```

Supplementary Figure 1 (Related to Figure 1). Chemical shift index analysis. Secondary structures are determined, using the web server “CSI 3.0”, by comparison of the assigned backbone NMR chemical shifts (Cα, CO, Cβ, N, Hα, NH) with reference chemical shift index.

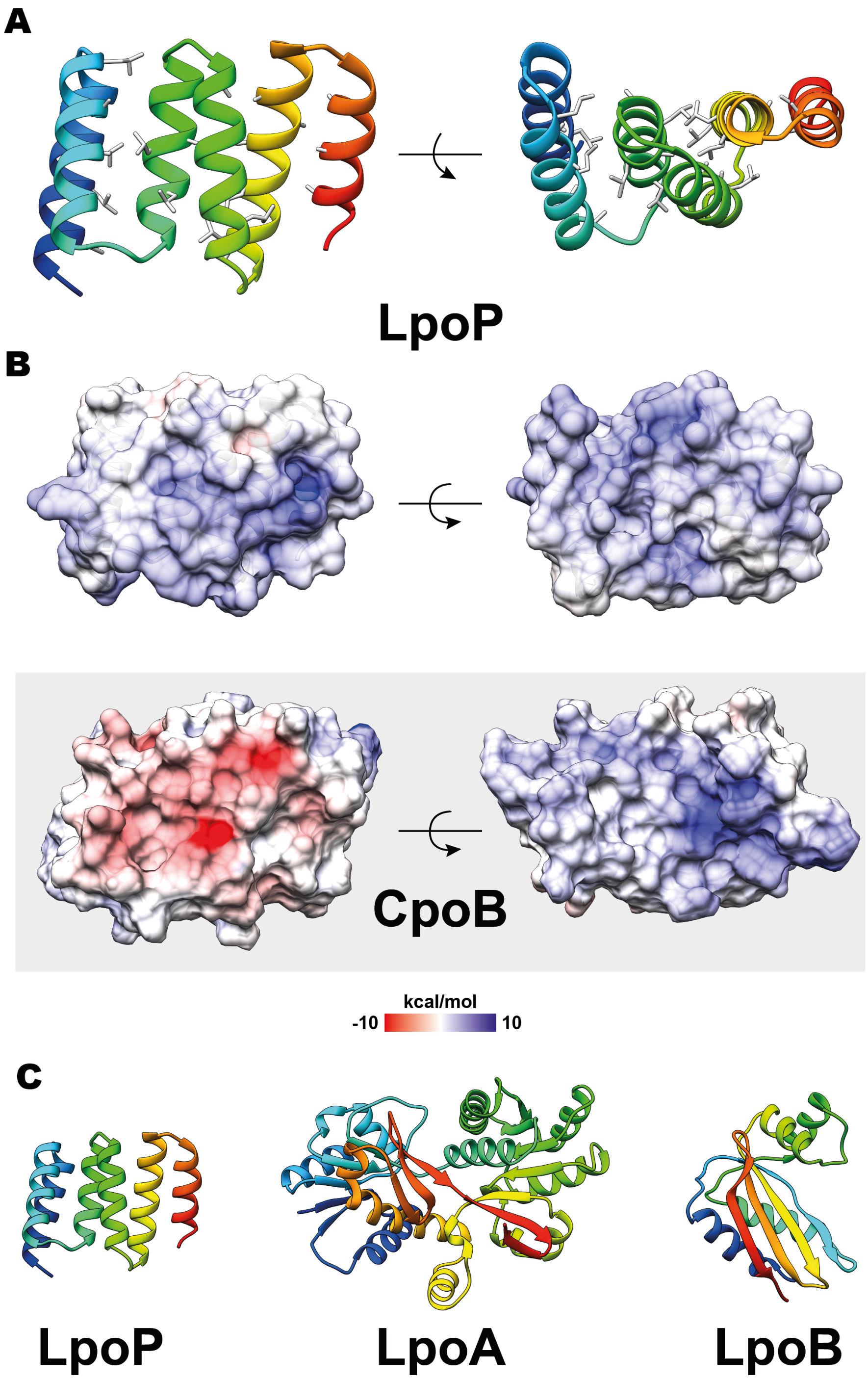

Supplementary Figure 2 (Related to Figure 2). Hydrophobic core of LpoP, electrostatic surface potential of LpoP and CpoB, and structural comparison of LpoP, LpoA and LpoB. (A) The inter-helical Leu and Ala hydrophobic contacts which stabilize the core of LpoP. **(B)** The electrostatic surface potential of LpoP and CpoB (PDB 2XEV), highlighting the differences between the electropositive LpoP and the electropositive and electronegative faces of CpoB. **(C)** The PBP binding domain of LpoP in comparison to that of LpoA (PDB 3CKM) and LpoB (PDB 4Q6Z).

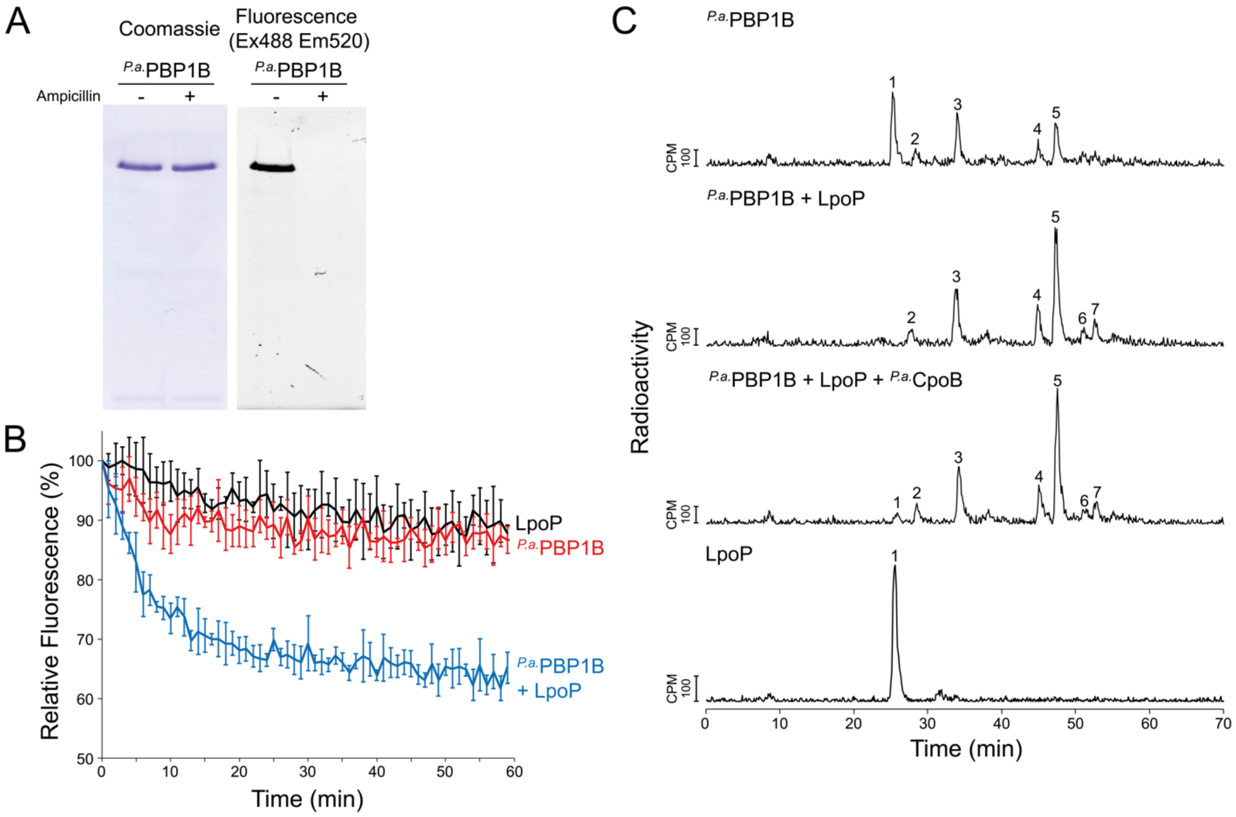

Supplementary Figure 3 (Related to Figure 3). Folding and activity of ^{Pa}PBP1b. (**A**) Fluorescence and coomassie stain images of an SDS-polyacrylamide gel on which Bocillin-bound ^{Pa}PBP1B was resolved. To ensure specificity of binding ^{Pa}PBP1B was incubated in duplicate at 37°C for 30 min, ampicillin was added to one of these duplicates (+). Bocillin was then added to both samples followed by further incubation at 37°C for 30 min. Specific binding of Bocillin shows the TPase domain, and presumably the entire protein, is correctly folded and functional. (**B**) GTase reaction rate data used to calculate relative rates shown in Figure 3B. Mean relative fluorescence (%), using the start-point as 100%, is plotted against time in minutes (min). The protein component of each reaction is shown next to the corresponding curve in the same colour. Polymerisation of the fluorescently labelled lipid II causes a decrease in fluorescence signal. Thus, the slope of these plots gives a relative measure of the GTase rate (n = 4). Data is the mean ± SD. Photobleaching of the fluorophore also occurs over time. (**C**) Representative examples of HPLC chromatograms for TPase/CPase data shown in Figure 3B. Peak 1; PentaP (remaining lipid II/glycan chain ends), peak 2; Tetra (GTase and CPase activity product), peak 3; Penta (GTase), peak 4; TetraTetra (GTase, TPase and CPase), peak 5; TetraPenta (GTase and TPase), peak 6, TetraTetraTetra (GTase, TPase and CPase), peak 7; TetraTetraPenta (GTase and Tpase).

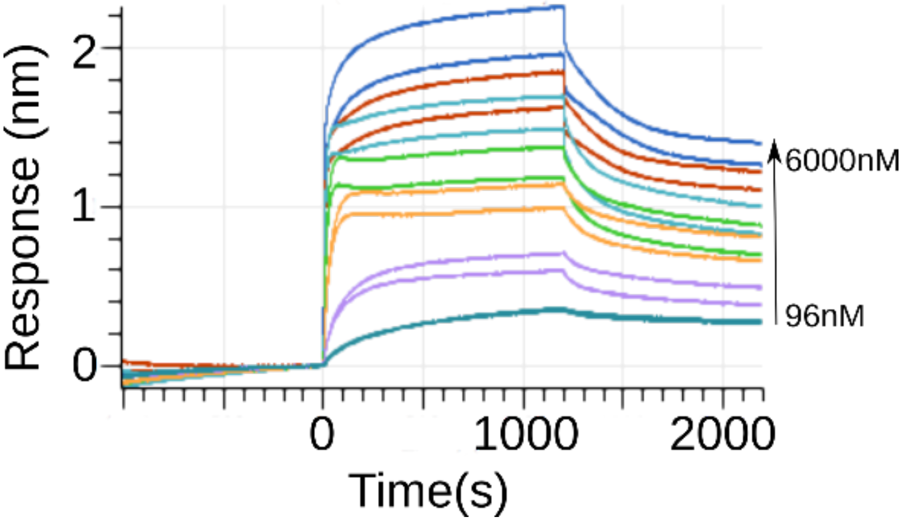

Supplementary figure 4 (Related to Figure 4). Real time BLI curves for LpoP and ^{Pa}UB2H. BLI sensorgrams recorded upon inserting ^{Pa}UB2H loaded biosensors into increasing concentrations of LpoP. Duplicates are represented with the same colour. Sensorgrams were subtracted from response of buffer alone and of the response of empty sensor tips dipped into different LpoP concentrations to remove unspecific binding contributions.

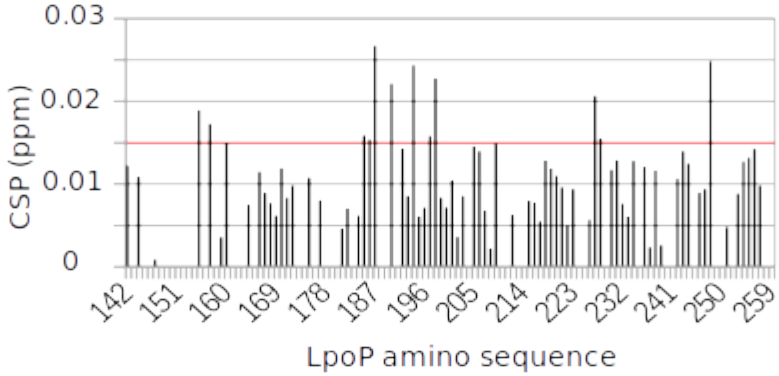

Supplementary Figure 5 (Related to Figure 4). Combined ^{1}H and ^{15}N chemical shift differences induced by ^{Pa}UB2H on LpoP. Residue-per-residue histogram of the chemical shift perturbations detected on the 1H,15N correlations of LpoP in a BEST-TROSY experiment collected after addition of His-^{Pa}UB2H in a 1:2 ratio.

Supplementary Table 1 (related to Star Methods). Crystallography data collection and refinement statistics.

	LpoP
**Data collection**	
Space group	P 1 2_1 1
Cell dimensions	
a, *b*, *c* (Å)	48.757, 154.584, 54.083
a, b, g (°)	90, 90.149, 90
Resolution (Å)	48.76 – 2.2 (2.279 - 2.2)*
R_{merge}	0.055 (0.372)
I / s*I*	13.16 (3.12)
Completeness (%)	98 (99)
Redundancy	3.2 (3.2)
**Refinement**	
Resolution (Å)	2.2
No. reflections	129749 (12773)
R_{work} / R_{free}	19.42 (26.90) / 22.99 (29.54)
No. atoms	
Protein	6210
Ligand/ion	85
Water	144
B-factors (Å^2)	
Protein	41.6
Ligand/ion	59.5
Water	42.3
R.m.s deviations	
Bond lengths (Å)	0.01
Bond angles (°)	0.99

*Values in parentheses are for highest-resolution shell.
